# Smoking Cessation Support in Social and Community Service Organizations: Potential Activities, Barriers, and Facilitators

**DOI:** 10.1093/ntr/ntae004

**Published:** 2024-01-09

**Authors:** Judith E M Visser, Andrea D Rozema, Anton E Kunst, Mirte A G Kuipers

**Affiliations:** Department of Public and Occupational Health, Amsterdam Public Health Research Institute, Amsterdam UMC, University of Amsterdam, Amsterdam, The Netherlands; Tranzo Scientific Center for Care and Wellbeing, Tilburg School of Social and Behavioral Sciences, Tilburg University, Tilburg, The Netherlands; Department of Public and Occupational Health, Amsterdam Public Health Research Institute, Amsterdam UMC, University of Amsterdam, Amsterdam, The Netherlands; Department of Public and Occupational Health, Amsterdam Public Health Research Institute, Amsterdam UMC, University of Amsterdam, Amsterdam, The Netherlands

## Abstract

**Introduction:**

Social and Community Service Organizations (SCSOs) are a potential setting to reach and support people with a low socioeconomic position who smoke, yet smoking cessation is not widely supported by SCSO professionals.

**Aims and Methods:**

This study aims to identify SCSO professionals’ (1) potential activities to support smoking cessation and (2) barriers and facilitators in undertaking these activities. Between July and November 2022, semi-structured interviews were conducted with 21 professionals recruited through SCSOs in Amsterdam North, including participation workers, welfare workers, parent and child counselors, budget coach, debt counselor, welfare work, community sports, and community center coordinators. Data were analyzed using a thematic approach.

**Results:**

Eight activities were identified that could support the client either directly (ie, recognizing smoking clients, discussing smoking and smoking cessation, referring clients, providing smoking cessation counseling, offering help around services) or indirectly (ie, collaboration with relevant network partners, implementing smoke-free environments, enhancing professional skills). Various barriers and facilitators were identified related to the (1) client and their environment (ie, clients’ readiness and social environment), (2) interaction between professional and client (ie, topic sensitivity), (3) professional (ie, professional is non-smoker, knowledge, and self-efficacy), (4) professionals’ work environment (ie, necessity, responsibility, priority, and time), and (5) smoking cessation services (ie, availability of appropriate services and referral process).

**Conclusions:**

There is potential for SCSO professionals to support smoking cessation, but several barriers hinder their efforts. To address these barriers, it is essential to take into account the factors that SCSO professionals believe facilitate the provision of smoking cessation support.

**Implications:**

This study provides insight into how the potential of SCSOs in Amsterdam North to support smoking cessation efforts among people with a low socioeconomic position can be harnessed. Barriers were found at multiple levels (client, professional, client-professional interaction, and organizational) and these findings imply that stakeholders across these levels will need to prioritize smoking cessation to facilitate and stimulate SCSO professionals in supporting smoking cessation. A concrete action would be to offer SCSO professionals additional training in conversational skills to discuss smoking. As a prerequisite, easily accessible and suitable smoking cessation services should be available in the neighborhood.

## Introduction

Cigarette smoking is the number one cause of morbidity and mortality.^1,2^ Prevalence rates continue to be significantly higher among those with lower socioeconomic position (SEP) than among those with higher SEP.^3,4^ This higher prevalence among people with lower SEP is an important contributor to socioeconomic inequalities in mortality and health in European countries.^5,6^ Smoking inequalities are also large in the Netherlands^7,8^ where in 2022, 23.2% of people with a low SEP were current smokers compared to 14.5% of people with a high SEP.

People with a low SEP may find quitting more difficult because of a variety of factors, including lack of social support for smoking cessation, low motivation to quit, stronger smoking addiction, psychological differences such as lower self-efficacy levels, and sensitivity to tobacco industry marketing.^9–11^ Moreover, people with a low SEP have been found to have less access to appropriate smoking cessation support that are generally the most effective.^9,10^ Effective strategies to reach and support people with a low SEP in smoking cessation are therefore important to reduce disparities in tobacco use.^9,10^

Social and Community Service Organizations (SCSOs) may be a potential new setting to reach and support smoking people with a low SEP. In the Netherlands, these are both municipal and non-municipal organizations that provide services and facilities in the areas of work, participation, self-reliance, well-being, and youth, with the main goal of enhancing quality of life and to enable citizens to fully participate in society.^12^ SCSOs have potential advantages as settings for undertaking various activities aimed at supporting smoking cessation, both activities that directly and indirectly support the client. SCSO professionals have existing established contact with lower SEP smokers and are experienced in working with this target group.^13^ Furthermore, professionals are in the position to provide personalized support and are ideally placed to address smoking in a holistic manner alongside other issues that hinder smoking cessation.^11,13–15^

A few studies on smoking cessation in SCSOs have been carried out in Australia and the United States. One study shows that professionals are willing to undertake certain activities to support clients in smoking cessation, such as asking about and recording the client's smoking status and providing information, brief advice, and referral.^14^ Previous studies have also identified some general barriers faced by SCSO professionals in undertaking these activities.^14,16^ For example, professionals do not see smoking cessation as a priority compared to other problems, such as homelessness, and drug and alcohol use.^14,16^ In addition, professionals experience a lack of specific knowledge, confidence, and skills to address smoking with their clients.^14,16^

Although SCSOs may be a promising setting for smoking cessation support in Australia and the United States, the international literature does not include studies from European countries on SCSOs as settings for smoking cessation support. Therefore, it is uncertain whether in European countries these services have the potential to provide support and whether the barriers are similar to those elsewhere.^17^ Moreover, in previous research, it remains unclear which specific activities professionals believe may be sufficiently important and feasible to incorporate into daily practice. By identifying these activities, and their barriers and facilitators, this study aims to contribute evidence to understand how SCSOs may be optimally utilized in smoking cessation support.

## Methods

### Design

We performed a qualitative study in which we conducted 21 semi-structured interviews with professionals from nine different SCSOs. Interviews are particularly useful in this study as they give insight into participants’ experiences, perceptions, and opinions regarding the provision of smoking cessation support.^18^

### Sample and Recruitment

To be eligible for this study, participants had to be employed as professionals in a SCSO, in positions such as socio-cultural or social worker, debt relief worker, activity supervisor, consultant, and project manager.^19^ In addition, SCSOs had to be located in Amsterdam North, a district characterized by persistent poverty and a relatively high prevalence of smoking.^20,21^ Managers from SCSOs who were not in contact with clients were not included.

A network analysis was performed to recruit and select all involved study participants, as this provided a clear overview of SCSOs in Amsterdam North. First, SCSO professionals based in Amsterdam North from our existing contacts were contacted via email and asked to complete a questionnaire about their collaborations with other SCSO professionals in Amsterdam North. Next, SCSO professionals who emerged from the questionnaire were invited to complete the same questionnaire, and so on. Finally, SCSO professionals who emerged from the network analysis were invited to participate in this study (*n* = 67). The response rate was 31% (*n* = 21). Most professionals who were invited, but did not participate, were either not interested in the topic or did not have time for an interview.

### Procedure

The data collection took place between July and November 2022. Interviews were conducted by the first author (JV). Most professionals were interviewed at their place of work, in local community centers (*n* = 14), and some professionals were interviewed through Microsoft Teams (*n* = 7). All participants received both oral and written information and signed informed consent. Interviews lasted 36 min on average (SD = 6; range 19–46). Before the interview, each participant completed a short questionnaire. After the interview, participants received a gift voucher of 30 euros. The interviews were audio-recorded and transcribed verbatim.

The study was guided by qualitative description methodology and reported using the COREQ checklist.^22^

### Instruments

The previously mentioned short questionnaire assessed socio-demographic characteristics gender, age, position within the organization, and number of years employed in this position.

The interview guide addressed topics including current and potential activities in smoking cessation support, and the barriers and facilitators in undertaking these activities (see Appendix A). In this study, current and potential activities could either directly or indirectly support the client. In the first part of the interview guide, participants were asked about the activities they were already undertaking to support clients in smoking cessation, followed by questions on the barriers and facilitators in undertaking these activities. In the second part of the interview guide, participants were asked about which activities they could potentially undertake, followed by questions on the barriers and facilitators for these potential activities.^14,16,23,24^

### Data Analysis

Transcripts were analyzed using a thematic approach of Braun and Clarke in MAXQDA 2022.^25^ First, four transcripts were independently double-coded, two transcripts by the first and last author (JV and MK), and two transcripts by the first and second author (JV and AR). Then, inconsistencies of the double-coded transcripts were discussed between the two coders of each transcript, and cases where consensus was not easily reached were discussed between all three coders. Next, the remaining transcripts were independently coded by one author (JV), as there was consistency in the previous parallel-coded transcripts. Subsequently, different codes were combined to form overarching sub-themes and themes. These themes were combined, refined, or separated. This process was iteratively checked by two authors (MK and AR) and disagreements were discussed with the first author (JV) to ensure that data were adequately categorized. The third author (AK) actively participated in the final stage of this process.

## Results

Background characteristics are provided in Table 1. Participants were 42 years old on average (range 25–65) and most were women (*n* = 14, 67.0%). They varied in their position within SCSOs, with the majority of participants working as welfare worker or participation worker (see Appendix B for a description of each position).

**Table 1.** Demographic Characteristics of SCSO Professionals

	N	%
Total	21	100
Age		
25–34	8	38.1
35–44	5	23.8
44–54	2	9.5
>55	6	28.6
Gender		
Female	14	67.0
Male	7	33.0
Years employed in the position		
0–3	12	57.0
4–7	6	29.0
8–11	0	0.0
>12	3	14.0
Position		
Participation worker	7	33.3
Welfare worker	4	19.0
Coordinator welfare work	2	9.5
Community center coordinator	2	9.5
Parent and child counselor	2	9.5
Budget coach	1	4.8
Debt counselor	1	4.8
Community sports coordinator	1	4.8
Project leader digital volunteer	1	4.8
Consultant volunteer work	1	4.8

### Potential Activities in Providing Smoking Cessation Support

All activities can be found in Table 2 and are discussed below.

**Table 2.** Professionals’ Potential Activities in Providing Smoking Cessation Support

Theme	Sub-theme	Some professionals do not undertake the activity^a^	Some professionals do undertake the activity ^a^	Professionals do see the activity as an opportunity
1 Recognizing smoking clients	Asking about smoking status	x	x	x
	Identifying smoking clients		x	x
2 Discussing smoking and smoking cessation	Discussing smoking	x	x	x
	Discussing the consequences of smoking		x	x
	Discussing smoking cessation		x	x
	Advising to quit smoking		x	x
	Motivating to quit smoking		x	x
3 Referring clients	Referral	x	x	x
	Referral to online support	x		
	Referral within the organization		x	
	Referral to external organization		x	x
	Referral to healthcare domain	x	x	x
4 Providing smoking cessation counseling	Providing smoking cessation counseling	x		x
5 Offering help around smoking cessation services	Organizing smoking cessation services		x	x
	Facilitating smoking cessation services		x	x
	Helping the client on the background		x	x
6 Collaborating with relevant network partners	Collaborating with parties	x	x	x
	Engaging local residents			x
	Linking clients to experts by experience			x
7 Implementing smoke-free environments	Making the organization smoke-free			x
8 Enhancing professional skills	Expertise promotion through training		x	x
	Expertise promotion in team meetings			x

^a^Resulting from semi-structured qualitative interviews. As not all activities were discussed with each participant, not all activities are marked in columns 3 or 4.

#### Recognizing Smoking Clients

To recognize smoking clients, professionals’ approach usually consists of direct observation of the client's smoking or asking the client about their smoking status. Professionals mentioned that they sometimes actively aim to recognize smoking in clients, but this is not standard practice. However, they see opportunities to make this part of the intake procedure. *“I ask about clients’ family circumstances, their social network, financial matters, housing. When I ask about their health, it would just be a simple question: Do you smoke?”*

#### Discussing Smoking and Smoking Cessation

Professionals report that actions to discuss smoking and smoking cessation can take various forms, including to initiate a conversation about smoking and its consequences, advise clients to quit and motivate them to make different choices in smoking behaviors, such as setting a quit date or smoking fewer cigarettes. In addition, they inform clients about smoking cessation services. Currently, professionals perform this activity variably, but they see opportunities to make it part of their tasks. *“The client does have some motivation, but the sense of urgency to quit is often not present. As a caregiver, it could be our task to promote that urgency.”*

#### Referring Clients

Professionals who refer clients to smoking cessation programs refer to programs within the organization, to external organizations, or to the healthcare domain, such as the general practitioner or the hospital. Some professionals already refer clients to smoking cessation services and others do not, but they intend to do so. *“Similar to how we direct people with weight-related concerns there [to specialized programs], we might think: Oh, that gentleman or that lady wants to get rid of it [smoking]. Well, go there [to a program], talk to someone. Then we would serve as a referral function. We won't manage that internally.”*

#### Providing Smoking Cessation Counseling

Professionals who provide smoking cessation counseling offer individualized guidance to assist clients in quitting smoking. Most professionals mentioned that they do not provide smoking cessation counseling yet, but they are willing to do so in certain circumstances. *“Providing support myself could be beneficial when we sense that clients will not take the step to smoking cessation services or services are not available.”*

#### Offering Help Around Smoking Cessation Services

Professionals who offer help around smoking cessation services provide ongoing support to clients throughout their journey to quit smoking, without providing smoking cessation counseling themselves. In addition, they facilitate smoking cessation services, such as offering a location or arranging smoking cessation trainers. Professionals indicated that they offer help around smoking cessation services every now and then, and there is potential to undertake this activity more often. *“Wedon't organize anything during Stoptober yet, but it would be perfect for a community center since we organize events every theme week. It would be really fun to facilitate a smoking cessation event in the neighborhood in a playful way.”*

#### Collaborating With Relevant Network Partners

Relevant network partners are existing contacts in the neighborhood with whom professionals collaborate to promote smoking cessation (ie, general practitioners, local residents, and other SCSOs). For instance, professionals from different SCSOs conduct brainstorming sessions to plan local events aimed at promoting healthy lifestyles among local residents, including smoking cessation. Additionally, professionals from the SCSO help connect local residents who have successfully quit smoking with those who wish to quit, enabling them to exchange their experiences. Currently, professionals work with relevant network partners on smoking cessation irregularly, but they indicated that this activity has potential. *“I would spend more time involving health ambassadors. These are local residents who help other local residents. For example, with healthy grocery shopping. The theme of smoking would also fit in very nicely.”*

#### Implementing Smoke-Free Environments

When implementing smoke-free environments, professionals encourage SCSO organizations to implement smoke-free zones around their buildings. Some professionals indicated that many people smoke in front of the entrance and that there are no smoke-free zones yet, but they see opportunities for their organization to introduce this. *“I think it is good that we try to set an example [with smoke-free zones around the building], to activate residents to think about it.”*

#### Enhancing Professional Skills

Professionals who actively enhance their skills participate in training sessions or team meetings in which they increase their skills to discuss smoking cessation and expand their knowledge of available smoking cessation services. While some professionals are enhancing their skills in providing smoking cessation support, others do not. Nevertheless, the latter group also indicated that enhancing their skills would be beneficial. *“I’m curious about how colleagues or other staff approach such a conversation [a conversation about smoking with a client]. I would like to make it a topic in our meetings or in a larger gathering.”*

### Barriers and Facilitators in Undertaking Smoking Cessation Activities

A total of 12 themes were identified that influence whether or not SCSO professionals undertake smoking cessation support activities (Table 3). Professionals consider most themes facilitators if present and barriers if absent.

**Table 3.** Barriers and Facilitators in Undertaking Smoking Cessation Activities

Level	Theme	Barriers	Facilitators
		Sub-theme	Sub-theme
**Factors related to the client and their environment**	1 Clients’ readiness	1.1 Client is not ready to quit 1.2 No request for help from the client	1.1 Client is ready to quit smoking 1.2 Client is open for discussion
	2 Social environment	2.1 Impact client's social environment	
**Factors related to the interaction between professional and client**	3 Topic sensitivity	3.1 Negatively affects the relationship 3.2 Professionals do not want to appear interfering 3.3 Client’s own choice 3.4 Smoking has benefits for the client 3.5 Smoking cessation is complex	3.1 Relationship of trust with a client 3.2 Professional is aware how to properly deal with a client
**Factors related to the professional**	4 Professionals’ non-smoking status	4.1 No understanding of smoking client 4.2 Non-smoking professional does not feel taken seriously	
	5 Knowledge	5.1 Lack of knowledge to provide smoking cessation support 5.2 Lack of knowledge of smoking cessation services	5.1 Knowledge to provide smoking cessation support 5.2 Knowledge of smoking cessation services 5.3 Knowledge about smoking
	6 Self-efficacy		6.1 Feeling confident in providing smoking cessation support
**Factors related to the professional's work environment**	7 Necessity	7.1 Lack of attention from the organization 7.2 Lack of attention from the municipality 7.3 Professional has no sense of urgency 7.4 Lack of attention from education 7.5 No sense of urgency in the healthcare domain	7.1 Attention from the organization 7.2 Attention from the municipality 7.3 Professional has sense of urgency
	8 Responsibility	8.1 Not part of professionals’ packages of tasks	8.1 Part of professionals’ packages of tasks
	9 Priority	9.1 Smoking cessation is not a priority of the professional 9.2 No request for help from a healthcare domain	9.1 Smoking cessation is a priority of the professionals 9.2 Smoking cessation is a priority of the municipality 9.3 Smoking cessation is a priority of the organization
	10 Time	10.1 Lack of time professional 10.2 Lack of time organization 10.3 Lack of time healthcare domain 10.4 No time frame from municipality	10.1 Time to provide smoking cessation support
**Factors related to smoking cessation services**	11 Availability of appropriate smoking cessation services	11.1 Smoking cessation services insufficient 11.2 Professional does not believe in services 11.3 Quantity of smoking cessation services not appropriate 11.4 Client does not believe in services	11.1 Smoking cessation services sufficient 11.2 Smoking cessation services available 11.3 Professional do believe in services
	12 Referral process	12.1 Step to smoking cessation services is a threshold	12.1 Step to smoking cessation services is no threshold

#### Factors Related to the Client and Their Environment

First, professionals indicated that clients’ social environment creates a sense of powerlessness, as fear of negative reactions from family and friends discourages clients from quitting smoking. *“People often find it challenging to admit to others that they may want to quit. In certain groups, individuals are sometimes ridiculed when they express a desire to stop smoking.”* Second, professionals mentioned clients’ readiness as barrier to undertaking activities in support. They indicated that clients are often not ready to quit as they deal with many other social issues, such as financial, relational or physiological problems and clients feel like they are unable to quit smoking at the same time as dealing with these issues. *“In fact, every client says: ‘I’m under too much stress right now...this happened and now that’. Stress is actually the factor in the entire group that makes people think they are not able to quit smoking right now.”* In addition, professionals believe that many clients have no desire to quit as they do not ask for help to quit smoking.

Conversely, professionals cited clients’ readiness as a facilitator when present. When clients’ readiness to quit is clear (ie, asks for help to quit smoking), professionals mentioned that they are more inclined to provide support. *“When they say something like: ‘I’m thinking about quitting’. Those are the words I need to hear to say: ‘we can help you’.”*

#### Factors Related to the Interaction Between Professional and Client

Professionals indicated that the topic sensitivity was a challenge to provide support. Professionals are apprehensive to harm the relationship of trust with their client, as they experience a fine line between “discussing smoking” and “judging a person who smokes.” *“Idon't thinkit's good for the relationship of trust with a client if you immediately start talking about the things someoneisn't doing right.”*

On the other hand, topic sensitivity was not only seen as a barrier, but also discussed as facilitating. Thanks to the relationship of trust, professionals were able to discuss sensitive topics, such as quitting smoking. *“You need to create a certain level of safety before confronting them too much. Otherwise, people might leave, stop staying in touch, andyou'll lose them.”* Moreover, professionals are trained to interact with clients professionally, for example by remaining neutral, so these skills enable professionals to prevent clients from feeling pushed or judged.

#### Factors Related to the Professional

First, professionals’ non-smoking status was perceived as a barrier, as they do not have much experience with smoking and smoking cessation. Consequently, professionals expressed concern that they cannot fully empathize with clients who smoke and fear that clients may feel misunderstood. *“I actually see it more as a barrier that Idon't smoke, because I feel like there is a distance between me and my client.”* Second, professionals indicated that they lack the knowledge of how to effectively discuss smoking as well as the knowledge of available smoking cessation services. *“I’m by no means an expert in this field. I’m simply sharing what I know from general knowledge about quitting smoking.”*

On the other hand, they cited knowledge as facilitating when present. *“How can we make the target group see the added value of quitting smoking? I think that is very interesting for us to know.”* According to professionals, possessing knowledge about smoking cessation may increase their self-efficacy, a factor that was also mentioned as a facilitator for undertaking activities in smoking cessation. *“When you feel competent, it also reduces the threshold to address such topics [asking about clients’ smoking status].”*

#### Factors Related to Professionals’ Work Environment

Professionals mentioned municipality awards grants to SCSOs to work on certain focus areas, and smoking cessation support is not included as a focus area. As a result, there are no agreements in organizations about professionals’ role in smoking cessation support, so they feel no necessity or responsibility to support smoking cessation of clients. *“Because it’s not top of mind, especially in SCSOs, it has received less attention from me.” “It’s not within our job description. That may sound a bit odd because our job descriptionisn't that straightforward; it covers a wide range of things, butit's not a basic thing we do, I think.”* Consequently, professionals do not consider smoking cessation as a priority. They also experience a lack of priority for smoking cessation, because they prioritize stabilizing the client's situation. *“If you are in debt or you are overweight and you are experiencing discomfort, we can certainly talk about smoking, butthat's not going to work because those other aspects really require priority.”* They believe that clients are unable to quit smoking at the same time as dealing with other issues, and professionals often consider these issues more important. As they do not treat it as a priority and state that it is not part of their assigned tasks, professionals are more likely to devote time to the defined focus areas and this leaves limited time to simultaneously work on smoking cessation support. *“Those 3 or 4 extra conversations that are sometimes needed to motivate someone to quit an addiction, well, I don't have the time for that because I don't get paid for it.”*

Conversely,  professionals cited all the previously mentioned barriers as facilitating when present. Professionals suggested that a sense of necessity, as well as responsibility, would contribute to undertaking smoking cessation activities. *“It’s awareness. That sounds a little lame, but I think that's where it starts.”“An additional role comes into play when problems in other life domains cause financial instability. If unhealthy behavior leads to an inability to manage debts, then you have an extra responsibility.”* Professionals also emphasized that it would be facilitating when they consider smoking cessation more as a priority and are given time to focus on it. *“If smoking is a priority above us [organization and government] or more often part of the conversation, it will definitely reach the professionals.” “If there is time for it, I would be open to it.”*

#### Factors Related to Smoking Cessation Services

First, professionals reported a lack of availability of appropriate smoking cessation services, including services that meet clients’ needs, to be a barrier. *“When there are no services nearby, it becomes challenging to enthuse clients. Then, I already know that people won't go.”* Of the services that exist, there is doubt about the effectiveness and even if effective services are available, the complicated referral process could also be a barrier as it often consists of multiple steps before a client reaches the services. *“It’s more difficult when you [speaking from a client's perspective] have to initiate contact [with the smoking cessation service]; you might even think: Never mind, it's not necessary.”*

Conversely, professionals mentioned the availability of appropriate smoking cessation services as a facilitator as it is reassuring to be able to offer something concrete when someone asks for cessation support. *“It is helpful to be able to refer someone when you notice someone is struggling and having difficulty quitting.”* In addition, a simple referral process was also mentioned as facilitating. *“If I register someone with [organization X], the organization itself contacts the client. That is easy, because in that way I don't have to ask the client to contact the organization, and I think that is less likely to happen.”*

## Discussion

### Key Findings

This study reveals that SCSO professionals see potential in a broad range of smoking cessation activities to incorporate into everyday practice that supports the client directly (ie, recognizing smoking addiction, discussing smoking and smoking cessation, referring clients, offering help around services, providing smoking cessation counseling) or indirectly (ie, collaboration with relevant network partners, implementing smoke-free environments, enhancing professional skills). Professionals indicated various barriers and facilitators that influence whether or not they undertake these activities related to the client and their environment (ie, clients’ readiness and social environment), the interaction between professional and client (ie, topic sensitivity), professional (ie, professional is non-smoker, knowledge, and self-efficacy), professionals’ work environment (ie, necessity, responsibility, priority, and time), and smoking cessation services (ie, availability of appropriate services and referral process). Professionals cited most barriers as facilitators when they were present.

### Strengths and Limitations

A strength of our study is that the inclusion of professionals from different organizations with various positions provided a heterogeneous group of SCSO professionals, resulting in a rich and detailed dataset. In addition, in comparison to previous research, we adopted a broad understanding of smoking cessation support, allowing us to explore a broader range of activities that professionals believe to be important and feasible enough to incorporate into daily practice. Nonetheless, this study also has a number of limitations that should be considered when interpreting the results.

First, selection bias may have affected the study. Total of 31% of the SCSO professionals who were approached agreed to participate. Possibly, professionals who were more active in smoking cessation support were more likely to be part of the study. The study included only two smoking professionals. Given that the smoking status of professionals appeared to influence their provision of support,^16,26^ findings may not be fully representative of the entire population of SCSO professionals.

Second, the study population comprises professionals working in organizations situated in Amsterdam North. The infrastructure of SCSOs in Amsterdam North may be different from the rest of Amsterdam.^26^ For instance, ten SCSOs in Amsterdam North have formed an integrated partnership based on a shared vision and common budgetary framework. Consequently, the findings may not be easily transferable to other geographical locations.

Lastly, as health promotion activities may have been viewed as socially desirable by both the participants and the interviewer, desirability bias may have occurred and this may have resulted in an overly optimistic overview of potential activities.^27^ This may limit the internal validity of the study. However, we did find many barriers, and participants did not report that all activities would be feasible, indicating that they did feel free to express more negative views.

### Interpretation of Findings

The results of this study show that professionals see the potential of various activities, but some smoking cessation support activities are not yet being undertaken. This is consistent with previous studies which have shown that SCSO professionals expressed a willingness to provide various types of smoking cessation support to clients.^14,16^ Additionally to these studies, our respondents also highlighted the potential of high-intensity activities, such as providing smoking cessation counseling or offering help around smoking cessation services. This may be attributed to the increasing role of SCSOs in the Netherlands in promoting healthy lifestyles.^28–30^ The objective of SCSO professionals is to support individuals in attaining meaningful and satisfying lives.^11^ Traditionally, their focus is primarily on addressing the psycho-social aspects of individuals within their environment, rather than their physical well-being. However, the involvement of SCSOs in addressing health issues is increasing.^29,30^ As a result, SCSO professionals may be more willing to provide intensive support to improve their clients’ physical well-being.

We found that smoking is not perceived as a priority at the municipal level and there are no agreements in organizations about professionals’ role in smoking cessation support. The lack of organizational guidelines for practices may explain why professionals do not provide standard support for smoking cessation. Previous studies show that implementing guidelines for SCSO professionals to routinely ask questions about tobacco use encourages them to provide cessation support to those who are found to be interested in quitting.^16,17,24^ Once organizational guidelines are implemented, they tend to be sustained over time without the need for ongoing intervention programs.^31^ From the healthcare sector, we know that several factors influence professionals’ adherence to guidelines, such as the simplicity of the guidelines and professionals’ awareness of their existence.^32^

Our study found that professionals prioritize stabilizing the client's situation over smoking cessation efforts. They believe that smoking may be necessary for maintaining stability in the client's circumstances. This finding aligns with previous research about professionals who discourage clients from quitting smoking. Professionals perceive smoking as an effective coping mechanism available to clients who are stressed and in crisis.^13,14^ However, evidence shows that smoking does not alleviate stress but increases the level of stress.^33^ The apparent relaxation effect experienced by people who smoke is a result of the temporary relief from tension and irritability during nicotine withdrawal. Because of the lack of awareness regarding this aspect, professionals may mistakenly perceive that smoking cessation efforts interfere with their goal of helping the client, resulting in a lower priority given to smoking cessation support.

Our research findings suggest that enhancing the knowledge and self-efficacy of professionals could facilitate their ability to provide smoking cessation support. This aligns with prior studies on the effectiveness of retraining programs for SCSO professionals.^23,34^ Training programs for smoking cessation have demonstrated their effectiveness in enhancing the confidence and knowledge of professionals in delivering support for quitting smoking, resulting in increased engagement in cessation support practices. Such training encompassed a comprehensive program addressing smoking cessation support, including brief intervention skills based on the 5A’s approach (Ask, Advise, Assess, Assist, and Arrange), and nicotine replacement therapy.^23^

### Conclusions

SCSO professionals recognize the potential of providing support for smoking cessation and can identify a broader range of activities that extend beyond direct support. To utilize this potential, stakeholders at multiple levels will need to recognize the necessity of smoking cessation and to foster the development of professionals’ skills in providing smoking cessation support. As a prerequisite, easily accessible and suitable smoking cessation services should be available in the neighborhood.

## Supplementary material

Supplementary material is available at *Nicotine and Tobacco Research* online.

ntae004_suppl_Supplementary_Appendix_A

ntae004_suppl_Supplementary_Appendix_B

## Data Availability

Interview transcripts cannot be shared publicly because of ethical restrictions.
