# Supplementary material for: Smoking Cessation Support in Social and Community Service Organizations: Potential Activities, Barriers, and Facilitators
Source: Nicotine Tob Res. 2024 Jan 9;26(7):922–30. doi: 10.1093/ntr/ntae004 (PMC11190048; doi:10.1093/ntr/ntae004)
Supplement: ntae004_suppl_Supplementary_Appendix_A [file ntae004_suppl_supplementary_appendix_a.docx]

**APPENDIX A: Translated interview guide**

| **Introduction interview: information and questions** *(duration approximately 5 minutes)* | | | | |
| --- | --- | --- | --- | --- |
| **Introduction** | | - Glad you would like to participate in this study and make time for it - Introduce yourself: 'I am working as a researcher, conducting this research commissioned by Amsterdam UMC in collaboration with Tranzo, Tilburg University.' - Stress confidentiality of the research! | | |
| **Explanation of the aim** | | - The study aims to identify the potential activities of SCSO professionals in smoking cessation and the factors that influence undertaking these activities. | | |
| **Participants** | | - Participants will be interviewed between July and November 2022. | | |
| **Topics** | | The interview consists of the following components:   - Professionals' perceptions of their role in promoting healthy behaviors/ smoking cessation - The current activities of professionals in smoking cessation support - The potential activities of professionals in smoking cessation support - The barriers and facilitators influencing the provision of smoking cessation support | | |
| **Duration of the interview** | | - Approximately between 30 and 60 minutes | | |
| **End of introduction** | | - If questions are not clear, please let us know. - Do you have any questions for now? | | |
| **Confidentiality** | | - Requesting permission to make audio recordings so that we can fully elaborate on the data at a later time. These recordings will be kept in a secure environment within the Amsterdam UMC, location AMC. The data from the interviews will then be analyzed pseudonymously. - Recorder on - Ask respondent for permission/informed consent when recorder is on, e.g., 'Can you confirm that you would like to participate in the study and consent to the recording of this interview?' Respondent's response: 'Yes' | | |
| **Core of the interview** | | | | |
| **Start** | | | - We will now start the interview | |
|  | | |  | |
| 1. We start with the recording (icebreaker) | | | **Perceptions of professionals regarding the role in promoting healthy behavior and smoking cessation**   - To what extent do you view it as your role to promote healthy behavior among your clients? - Why do you consider it your role/ why not? - To what extent do you view it as your role to change your client's smoking behavior? - Why do you view it as your role or not to change your client's smoking behavior?   Optional:  *If you do not perceive it as your role to promote healthy behavior and/or smoking cessation...*   - To what extent do you view it as your organization's role to promote healthy behavior among clients? - Why do you consider it the organization's role/why not? - And changing smoking behavior? - Why do you consider it the organization's role/ why not? - Why do you believe your organization does or does not have a role in changing your client's smoking behavior?   *Within your organization, if you do not see it as your role to promote healthy behavior and/or smoking cessation...*   - According to you, who is better suited for this role? - Why?   *Interim and concluding summary; question: Have I correctly understood that...?* *(Duration approximately 5/10 minutes)* | |
| 1. We will now delve deeper into the activities you undertake | | | **Current activities of professionals in smoking cessation support**   - To what extent do you currently contribute to promoting your client's healthy behaviors? - Why yes/no? - How do you speak to your clients about this? - What activities do you carry out for this purpose? - Why? - How do you experience this? - To what extent do you currently contribute to improving your client's smoking behavior? - Why yes/no? - How do you speak to your clients about this? - What do you already know about smoking cessation support?   *I see that you indicate doing x (from the questionnaire)*   - How is that going? - What do you think about that?   *You indicated that you undertake these activities and you said the following about this....*   - *In addition to this, are there any other activities you perform regarding smoking cessation?* - *Which *activities are these?* - *Why?* - *How do you experience this?*   **Per named activity   - What barriers did you experience in performing this activity? - Why? - What factors contributed to the performance of this activity? - What do you need in order to perform this activity well? - Why?   *Activities (allowing the participant to speak first, then giving options)   - Increasing motivation to stop smoking - Provide support to stop smoking - Offering a low-threshold smoking cessation program - Referring to primary care physician to quit smoking - Referring to outside smoking cessation support programs - Providing tips on smoking cessation resources such as leaflets, apps, websites - Giving a reward (money or other rewards) for smoking cessation - Recommending nicotine replacement devices (nicotine patches and lozenges) - Offering free nicotine substitutes (nicotine patches and lozenges)   **Barriers/promoting factors (let the participant speak for themselves first, then give options)   - See tackling smoking as a priority - Smoking status/ smoking history of a professional - Smoking cessation programs in the organization - tools for resources/referral outside organization - knowledge to provide support - Feel qualified to provide support - Confidence to approach clients   *Interim and concluding summary; question: Have I correctly understood that...?* *(Duration approximately 15/20 minutes)* | |
| 1. Then we will take a closer look at the potential activities | | | **The potential activities of professionals in smoking cessation support**   - To what extent are you open to do more in your clients' smoking cessation efforts? - Why yes/no? - What (more) could you do for your client with regard to smoking cessation support? - What activities could you do (more) for this purpose? - Why? - In what time frame could you carry this out? - Why?   Per named task   - What barriers do you anticipate in performing this activity? - Why? - What factors will facilitate the performance of this activity? - What do you need in order to perform this activity? - Why? - To what extent do you consider yourself the appropriate person to do this?` - Why yes/no? - Who else within the organizations?   *Interim and concluding summary; question: Have I correctly understood that...?* *(Duration approximately 15 minutes)* | |
| **Completion of interview** (Duration about 5 minutes) | | | | |
| **Closing interview** | | | | - Is there anything else you would like to add to the conversation? - Are there things not yet covered that you feel are important to this study? - What did you think of the interview? |
| **Thanking for interview** | | | | - I would like to thank you for the interview and the time you were willing to give |
| **Additional questions** | | | | - If there are any questions, please contact me |
| **Stop recording** | | | |  |
|  | **Role and task** | | | |
| **Interviewer** | - Content leadership of conversation - Raises main questions (see guide) - Asks further questions if necessary (see guide) - Concluding and summarizing in between | | | |
