# Supplementary material for: Smoking Cessation Support in Social and Community Service Organizations: Potential Activities, Barriers, and Facilitators
Source: Nicotine Tob Res. 2024 Jan 9;26(7):922–30. doi: 10.1093/ntr/ntae004 (PMC11190048; doi:10.1093/ntr/ntae004)
Supplement: ntae004_suppl_Supplementary_Appendix_B [file ntae004_suppl_supplementary_appendix_b.docx]

**APPENDIX B**

| *Participation worker* | A participation worker is a professional who is tasked with facilitating and encouraging the active engagement and involvement in activities of individuals within a specific community. |
| --- | --- |
| *Welfare worker* | A welfare worker is a professional who is dedicated to providing assistance, support, and resources to individuals, families, and communities to enhance their overall well-being and quality of life. |
| *Coordinator welfare work* | A coordinator welfare work is a professional who holds a leadership position within an SCSO. This position involves overseeing and coordinating various aspects of welfare and social support programs to ensure effective service delivery and the well-being of individuals, families or communities in need. |
| *Community center coordinator* | A community center coordinator is a professional responsible for overseeing the operations, programs and activities of a community center to ensure that it serves as a center for community engagement, social interaction and the provision of essential services and resources. |
| *Parent and child counselor* | A parent and child advisor is a professional specialized in providing support and guidance to parents and their children in the areas of parenting and child development. |
| *Budget coach* | A budget coach is a professional who provides personalized guidance, education, and assistance to individuals or families in managing their finances effectively. The primary role of a budget coach is to help clients create and maintain a practical budget, make informed financial decisions, and achieve their financial goals. |
| *Debt counselor* | A debt counselor is a professional who specializes in providing guidance, support, and strategies to individuals or families who are facing financial challenges, particularly related to managing and repaying debts. The primary goal of a debt counselor is to help clients regain control of their finances, develop effective debt management plans, and work towards achieving a more stable and sustainable financial situation. |
| *Community sports coordinator* | A community sports coordinator is a professional who works to promote and organize sports and recreational activities within a local community. Their role involves bringing people together, fostering a sense of community, and encouraging physical activity through various sports programs and events. |
| *Project leader digital volunteer* | A Project leader digital volunteer is a professional who assumes a leadership role in coordinating and supervising a project in which volunteers are paired with local residents who, due to physical and/or psychological reasons, are homebound and wish to enhance their digital skills and require support in doing so. |
| *Consultant volunteer work* | A consultant volunteer work is a professional who provides expert guidance, advice, and support to organizations, groups, or individuals engaged in volunteer-related initiatives. |
